# Chemical changes of Angelicae Sinensis Radix and Chuanxiong Rhizoma by wine treatment: chemical profiling and marker selection by gas chromatography coupled with triple quadrupole mass spectrometry

**DOI:** 10.1186/1749-8546-8-12

**Published:** 2013-06-06

**Authors:** Janis Ya-xian Zhan, Wendy Li Zhang, Ken Yu-zhong Zheng, Kevin Yue Zhu, Jian-ping Chen, Pui-Hei Chan, Tina Ting-xia Dong, Roy Chi-yan Choi, Henry Lam, Karl Wah-keung Tsim, David Tai-wai Lau

**Affiliations:** 1 Division of Life Science and Center for Chinese Medicine, The Hong Kong University of Science and Technology, Hong Kong SAR, China; 2 Department of Chemical and Biomolecular Engineering, The Hong Kong University of Science and Technology, Hong Kong SAR, China; 3 Division of Biomedical Engineering, The Hong Kong University of Science and Technology, Hong Kong SAR, China

## Abstract

**Background:**

Angelicae Sinensis Radix (ASR) and Chuanxiong Rhizoma (CR) can be treated with wine to promote their biological functions in Chinese medicine. Both ASR and CR contain similar volatile chemicals that could be altered after wine treatment. This study aims to identify the differential chemical profiles and to select marker chemicals of ASR and CR before and after wine treatment.

**Methods:**

Chemical analyses were carried out by gas chromatography-triple quadrupole mass spectrometry (GC-QQQ-MS/MS) coupled with multivariate statistical analysis. Characterization of the compositions of essential oils was performed by automated matching to the MS library and comparisons of their mass spectra (NIST08 database). For ferulic acid, butylphthalide, Z-butylidenephthalide, senkyunolide A and Z-ligustilide, the mass spectrometer was operated in electron ionization mode, the selection reaction monitoring mode was used and an evaluation of the stability and sensitivity of the chromatographic system was performed for the tested extraction.

**Results:**

Principal component analysis (PCA) simultaneously distinguished ASR and CR from different forms. Ferulic acid, Z-butylidenephthalide, Z-ligustilide, butylphthalide and senkyunolide A were screened by PCA loading plots and can be used as chemical markers for discrimination among different groups of samples.

**Conclusion:**

Different chemical profiles of ASR and CR after wine treatment could be identified by GC-QQQ-MS/MS. The five marker chemicals selected by PCA, namely ferulic acid, butylphthalide, Z-butylidenephthalide, senkyunolide A and Z-ligustilide, were sufficient to distinguish between the crude and corresponding wine-treated forms of ASR and CR.

## Background

Angelicae Sinensis Radix (ASR; roots of *Angelica sinensis* (Oliv.) Diels; family Apiaceae; *Danggui* in Chinese) and Chuanxiong Rhizoma (CR; rhizomes of *Ligusticum chuanxiong* Hort.; family Apiaceae; *Chuanxiong* in Chinese) are adopted as drugs or dietary supplements in Asian countries. ASR can tonify blood and relieve amenorrhea, and can thus be used as a health food supplement for women [1-3]. CR is used to relieve headache, rheumatic arthralgia and coronary heart diseases [4-6].

Herbal processing is a crucial procedure for improving herbal functions [7-9]. Treatment with wine is one of the widely adopted methods, after which the treated herbs show enhanced blood circulation and accelerated drug delivery [10]. Wine treatment of ASR and CR alters the amounts of ferulic acid and total volatile oils [11-13], which are the pharmacologically active ingredients of both ASR and CR [14,15]. However, the specific changes of the volatile oils in ASR and CR after wine treatment, especially the biologically active chemicals such as butylphthalide, Z-butylidenephthalide, senkyunolide A and Z-ligustilidehave not been delineated. Since the crude and wine-treated forms of ASR and CR may show significant differences in their chemical compositions and clinical efficacy [9,16], identification of the different forms by chemical profiling methods would be a prerequisite for good quality control.

Some forms of ASR and CR are indistinguishable because of the similarities in their chemical compositions. Since their decoction or freeze-dried powder forms would cause technical problems if used in conventional chemical methods, sophisticated methods such as gas chromatography-triple quadrupole mass spectrometry (GC-QQQ-MS/MS) with high sensitivity and accuracy were adopted in this study to identify the chemical profiles of herbal extracts from ASR and CR. To our knowledge, this is the first study to analyze the changes in the chemical profiles of ASR and CR before and after wine treatment by GC-QQQ-MS/MS.

## Methods

### Chemicals, reagents and herbal materials

Ethyl acetate for GC analysis was purchased from Merck (Darmstadt, Germany). The chemical standards for butylphthalide, Z-butylidenephthalide and senkyunolide A were purchased from Weiqike Biotechnology Co. (Sichuan, China). Ferulic acid was obtained from Sigma (St. Louis, MO, USA). Z-Ligustilide was kindly provided by Prof. Pengfei Tu, Medical College of Peking University (Beijing, China). All chemical standards were tested and confirmed to show >98% purity based on their GC profiles and MS data. Fresh roots of ASR and fresh rhizomes of CR were collected in Gansu and Sichuan, China, respectively, as areas in which the best quality of the herbs can be guaranteed [17]. The identity of the herbs was confirmed by morphological authentication, and their voucher specimens (#09-11-02 for ASR and #09-09-20 for CR) were deposited in the Center for Chinese Medicine R&D, Hong Kong University of Science & Technology (Clear Water Bay, Kowloon, Hong Kong). All chemical reagents used in the study were of analytical grade. Individual samples were prepared from 1 kg of powdered herbal materials. The dried roots (50 g) were sliced, sprayed with 5 mL of yellow wine (Best Spirits, Zhejiang, China; 15–16% alcohol) and placed in an oven at 80°C for 90 min with flipping twice per hour to ensure standardized processing of ASR and CR. The protocol was conducted in accordance with our previous study [10].

### Herbal extraction

Ethyl acetate solution (25 mL) was added to 0.5 g of ground powders of ASR, CR, wine-treated ASR and wine-treated CR, respectively. Each mixture was then sonicated in an ultrasonic bath (240 W) for 20 min. In the second extraction, the residues from the first extraction were filtered, and the same extraction procedures were repeated. After centrifugation (Heraeus Megafue 1.0R; Waltham, MA, USA) at 2,500 × *g* and 4°C for 5 min, the supernatants were collected for GC-MS/MS analysis.

### GC-QQQ-MS/MS analysis

A 7000 GC/MS/MS series system (Agilent Technologies, Waldbronn, Germany) equipped with a 7890A gas chromatograph and GC-QQQ MassHunter workstation software (B.05.00.412; Agilent Technologies, Waldbronn, Germany) was used. Each extract was separated in an HP-5MS capillary column (250 μm × 30 m × 0.25 μm; Agilent Technologies, Waldbronn, Germany) with controlled temperature at 70°C in the initial stage, followed by elevation of the temperature to 280°C at a rate of 10°C/min. A pulsed splitless injection was conducted by injecting 1 μL of the sample extract. Helium was used as the carrier gas at a flow rate of 2.25 mL/min, and nitrogen was used as the collision gas at a flow rate of 1.5 mL/min. The spectrometer was operated in a full-scan electron-impact mode and the ionization energy was 70 eV. The inlet and ionization source temperatures were 250°C and 230°C, respectively. The solvent delay time was 3.5 min. The retention indices of all compounds were determined according to the Kovats method using *n*-alkanes as standards. The volatile flavor compounds were authenticated by comparing the mass spectra with the Kovats retention indices, NIST standard reference database (NIST 08) and other published databases of mass spectra. For the MS/MS analysis, suitable precursor ions and two product ions were selected for acquisition in the MRM mode of ferulic acid, butylphthalide, Z-butylidenephthalide, senkyunolide A and Z-ligustilide. The fragmentor voltage and collision energy values were optimized to achieve the greatest abundance of the detected compounds. The GC-QQQ MassHunter workstation software was applied for data acquisition and processing.

### Method validation

The linearity, sensitivity, precision, repeatability and accuracy of different analytes were determined to validate the analytical methods. For linearity, a calibration curve of each chemical compound was constructed from a range of working concentrations, and each line was plotted against six different concentrations. The limit of detection (LOD) and limit of quantification (LOQ) were used to determine the sensitivity. The lowest concentration of the working solution was diluted with ethyl acetate to obtain a series of appropriate concentrations for determination of the LOD and LOQ at signal-to-noise (S/N) ratios of 3 and 10, respectively. The assay precision was determined along with consideration of the intra-day and inter-day variations by analyzing standard solutions within 1 day (*n*=5) and on 3 consecutive days (*n*=6). For the repeatability test, five samples were prepared. A recovery test was used to determine the method accuracy. This was performed by adding the mixed standard solution into the herbal materials with known concentrations. The mixtures were then extracted and analyzed in triplicate as previously described. The recoveries were calculated using the following formula:

$$\text{Recovery}\;\left(\%\right)\;=\;100\;\times \;\left(\text{detected}\;\text{amount}\;-\right)\;/\text{amount}\;\text{spiked}.$$

The relative standard deviation (RSD) was used to describe the precision, repeatability and recovery.

### Statistical analysis

The peaks beyond the S/N ratios were labeled and manually integrated into chromatograms using the 7890A gas chromatograph and Chemstation software (B.03.01; Agilent Technologies, Waldbronn, Germany) to determine the differences in the volatile chemicals in the herbal materials after wine treatment. Principal component analysis (PCA) of the relative peak areas was performed using SPSS for Windows 16.0 software (SPSS Corporation, Armonk, NY, USA) to evaluate the discriminability of the peaks. Data were expressed as the mean ± standard deviation (SD) for *n*=3–8. Statistical tests were performed by one-way ANOVA. In the statistical analyses, differences were classed as significant for values of *P* < 0.05, and highly significant for values of *P* < 0.01 and *P* < 0.001.

## Results and discussion

### Validation of GC-QQQ-MS/MS methodology

**Figure 1:**
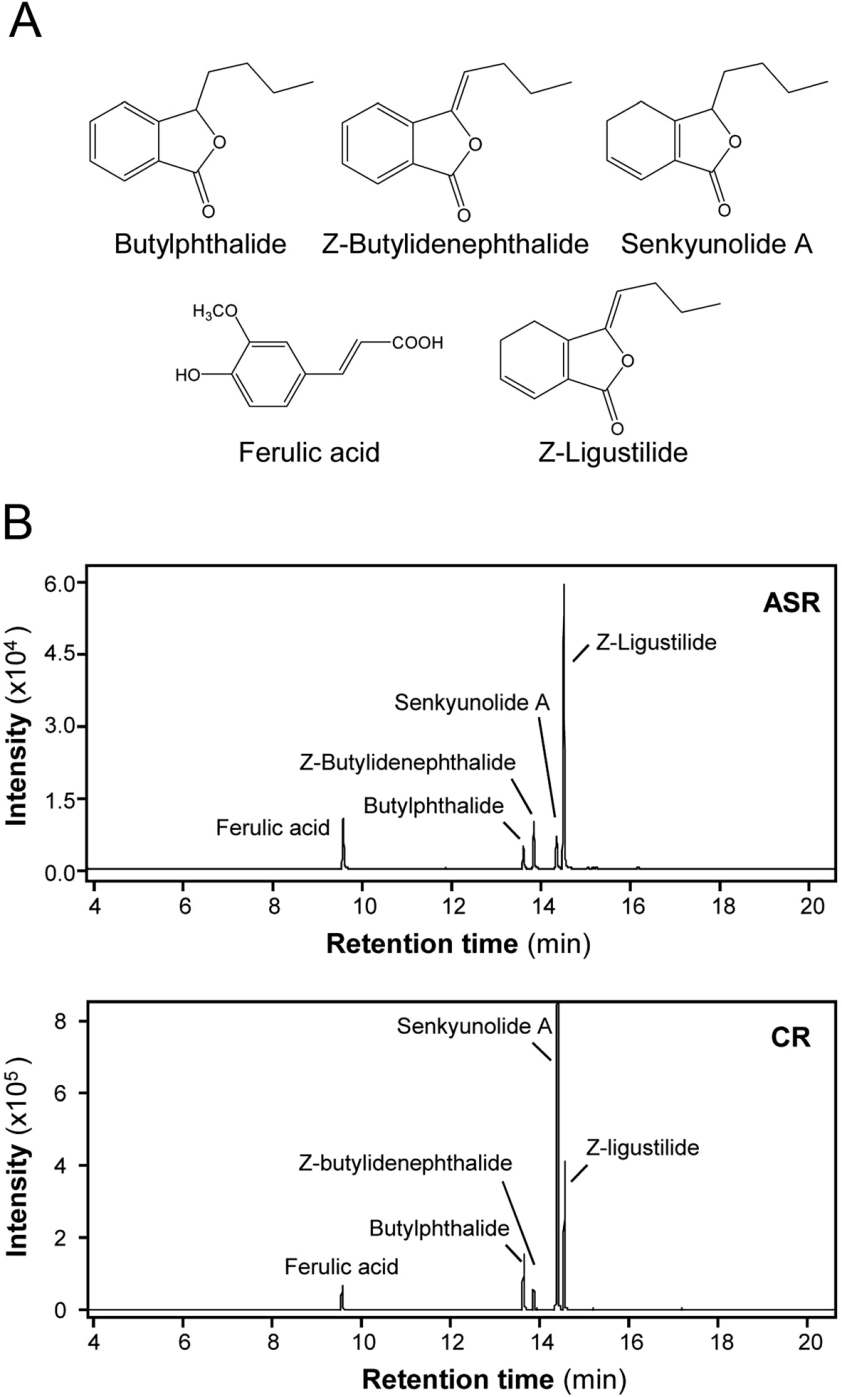
**GC-MS/MS MRM chromatograms of extracts from ASR and CR.** (**A**) Chemical structures of ferulic acid, butylphthalide, Z-butylidenephthalide, senkyunolide A and Z-ligustilide. (**B**) GC-MS/MS MRM chromatograms of ethyl acetate extracts of ASR and CR. Aliquots of 1 L were injected. The information for each peak is indicated. Representative chromatograms are shown (*n*=3).

Ferulic acid, butylphthalide, Z-butylidenephthalide, senkyunolide A and Z-ligustilide in ASR and CR were selected for detailed analyses, and their structures are shown in Figure 1A. The MS characteristics of the chemicals were also revealed (Additional file 1: Figure S1). Suitable precursor ions and two product ions were selected for acquisition in the MRM mode of ferulic acid, butylphthalide, Z-butylidenephthalide, senkyunolide A and Z-ligustilide (Table 1). The intensity and distinction of these marker chemicals were then calibrated with the product ions. Under these analytical conditions, the amounts of ferulic acid, butylphthalide, Z-butylidenephthalide, senkyunolide A and Z-ligustilide were successfully determined in the extracts of ASR and CR (Figure 1B).

**Table 1:** Mass spectra properties of the marker chemicals

**No.**	**Chemical**	**Formula**	**Molecular weight**	**Precursor ion **^**1**^	**Product ion **^**2**^	**Retention time (min) **^**3**^
1	**Ferulic acid**	C_10_H_10_O_4_	194.18	150	107	9.518
					135	
2	**Butylphthalide**	C_12_H_14_O_2_	190.24	133	51	13.682
					77	
3	**Z-Butylidenephthalide**	C_12_H_12_O_2_	188.22	159	103	13.922
					131	
4	**Senkyunolide A**	C_12_H_16_O_2_	192.25	107	77	14.453
					79	
*5*	**Z-Ligustilide**	C_12_H_14_O_2_	190.24	148	77	14.624
					105	

^*1*^The ion with the highest relative intensity was used as the precursor ion for quantification.

^*2*^Two product ions were used for the MRM analysis. The upper ion was used for quantitative analysis and the lower ion was used for qualitative analysis, which could guarantee the precision of the analytes.

^*3*^The retention time was determined by three different individual analyses (*n*=3).

Regarding the calibration of the marker chemicals, the regression equations of the five chemicals together with their LOD and LOQ values are shown in Additional file 2: Table S1. A mixed solution of the five analytes at various known concentrations was injected in triplicate, and the calibration curve of each analyte was plotted as the peak area against the concentration. The calibration curves demonstrated good linear regression with correlation coefficients of >0.990 in the test ranges. Using MS/MS analyses, the LOD and LOQ values were given for the ranges of 0.001–0.020 and 0.005–0.080 μg/mL, respectively. The overall intra-day and inter-day precisions were determined by analyzing the known concentrations of the five analytes. Solutions prepared from the herbal extracts of either ASR or CR were analyzed to test the repeatability. Variations were expressed as the RSD. The values of all intra-day and inter-day variations were found to be <5.0% (Additional file 3: Table S2). In addition, the validation studies showed that the assay had high reproducibility with an RSD of <5.0% (*n*=5). The average recoveries of the marker chemicals ranged from 95.58% to 98.75%, and their RSD values were all <5.0% (Additional file 3: Table S2). Therefore, the application of the GC-QQQ-MS/MS method in this study was precise, accurate and sensitive for simultaneous and quantitative evaluation of the marker chemicals in ASR and CR.

### Chemical variations of ASR and CR after wine treatment

The developed method was applied for quantification of the five chemical markers in ethyl acetate extracts of ASR or CR in both crude and wine-treated forms. The results are summarized in Table 2. In ASR, the amounts of ferulic acid, Z-butylidenephthalide and Z-ligustilide were altered after wine treatment, while the amounts of butylphthalide and senkyunolide A remained relatively constant. The amount of ferulic acid showed an increase of 40% compared with that in crude ASR (*P* = 0.023). In contrast, the yields of Z-butylidenephthalide and Z-ligustilide in wine-treated ASR were significantly reduced, and ~40% and ~25% lower than those in crude ASR (*P* = 0.035 and *P* = 0.021, respectively). After the wine treatment, the amounts of ferulic acid, butylphthalide and senkyunolide A in CR also varied significantly, while the amounts of Z-butylidenephthalide and Z-ligustilide remained relatively stable. The quantity of ferulic acid in wine-treated CR was 20% higher than that in crude CR (*P* = 0.036). In contrast, the yields of butylphthalide and senkyunolide A showed reductions of ~40% and ~30% (*P* = 0.047 and *P* = 0.003, respectively) compared with crude CR.

**Table 2:** Quantitative determination based on marker chemicals of Angelicae Sinensis Radix and Chuanxiong Rhizoma

**Chemical**	**Angelicae Sinensis Radix (mg/100 g)**		**Chuanxiong Rhizoma (mg/100 g)**	
	**Crude**	**Wine-treated**	**Crude**	**Wine-treated**
**Ferulic acid**	64.12 ± 8.03 ^1^	86.35 ± 5.12*	135.01 ± 7.70	161.47 ± 12.64*
**Butylphthalide**	2.21 ± 0.10	2.34 ± 0.21	7.66 ± 0.27	4.54 ± 0.16*
**Z-Butylidenephthalide**	21.73 ± 3.10	13.13 ± 1.35*	34.36 ± 0.17	33.90 ± 0.22
**Senkyunolide A**	23.53 ± 5.34	21.91 ± 2.04	140.55 ± 5.44	101.21 ± 3.18**
**Z-Ligustilide**	452.32 ± 16.38	326.06 ± 11.67*	839.53 ± 10.47	825.58 ± 9.82

^*1*^Values are expressed in mg/100 g of dried powder of crude herb, and represent the mean ± SD (*n*=3).

*P < 0.05, **P < 0.01, significant difference for crude extract versus wine-treated extract.

The major chemical changes of ASR and CR after wine treatment occurred in the volatile chemicals, such as essential oils, and therefore their amounts were compared between the crude and wine-treated herbal extracts. The relative amounts were expressed in terms of the percentage of the peak area in the wine-treated herbal extract to the total peak area of the untreated herbal extract, as generated from the GC analyses. A total of 56 chemicals from ASR or CR were selected for the comparisons (Table 3). Overall, 40 of the 56 chemicals were found in both ASR and CR, while six chemicals were unique to ASR and 10 chemicals were unique to CR. In wine-treated ASR, 11 chemicals were increased, 32 chemicals were reduced and two chemicals remained unchanged. Meanwhile, in wine-treated CR, seven chemicals were increased, 19 chemicals were reduced and the remaining chemicals were unchanged. As a result, wine treatment was proven to have more significant effects on chemical changes of ASR compared with those of CR.

**Table 3:** Components of the volatile chemicals in Angelicae Sinensis Radix and Chuanxiong Rhizoma

**No.**	**RI **^**1**^	**Components**	**RA **^**2 **^**(%)**				**No.**	**RI**	**Components**	**RA (%)**			
			**ASR **^**3**^	**WASR**	**CR**	**WCR**				**ASR**	**WASR**	**CR**	**WCR**
1	1083	3-Carene	-	-	0.06	0.08	29	1603	β-Humulene	0.12	0.26	0.14	0.12
2	1204	Sylvestrene	-	-	0.04	0.03	30	1628	Aromadendrene	-	-	0.05	0.05
3	1218	3-Ethyl-3-methylheptane	0.02	0.03	-	-	31	1640	Butylphthalide ^4^	3.25	2.98	8.70	7.26
4	1220	2-Methyl nonane	0.22	-	0.08	0.07	32	1645	Pentadecane, 8-heptyl-	0.29	0.17	-	-
5	1224	o-Cymene	0.69	0.19	0.05	0.05	33	1651	Z-Butylidenephthalide ^4^	7.79	6.15	3.28	3.12
6	1228	Limonene	0.63	0.31	0.04	0.04	34	1655	β-Eudesmol	0.13	0.09	0.81	0.55
7	1233	β-Pinene	3.63	1.69	0.25	0.29	35	1661	E-Butylidenephthalide	0.15	0.10	2.05	1.86
8	1246	4-Octanone	0.09	-	-	-	36	1675	Senkyunolide A ^4^	3.04	2.81	48.0	40.2
9	1256	β-Myrcene	1.06	0.42	0.07	0.10	37	1678	Muurola-4,11-diene	0.14	0.07	2.86	2.44
10	1260	Heneicosane	1.65	0.60	0.10	0.13	38	1683	Z-Ligustilide ^4^	48.7	40.7	25.5	25.1
11	1266	Ferulic acid ^4^	6.38	8.86	3.98	4.26	39	1687	1-Nonadecene	0.46	0.27	-	-
12	1285	β-Cedene	-	-	0.11	0.09	40	1692	Cryptone	0.58	0.33	0.03	0.02
13	1400	Tetradecane	0.13	0.22	0.02	0.03	41	1697	3,9-Diethyl-6-tridecanol	0.21	0.38	0.02	0.02
14	1408	Butanoic acid	-	-	0.03	0.02	42	1804	5-(2-Thienyl)-4-pyrimidinamine	0.19	0.33	0.03	0.02
15	1423	Caryophyllene	0.14	0.06	0.27	0.32	43	1809	γ-Eudesmol	0.27	0.18	0.10	0.08
16	1429	Pentyl benzene	0.37	0.22	0.02	0.02	44	1816	E-Ligustilide	8.05	6.52	0.48	0.45
17	1434	Eicosane	0.71	0.40	0.10	0.11	45	1849	6,7-Dihydroxyligustilide	0.12	0.08	0.13	0.08
18	1438	Di-tert-dodecyl disulfide	0.41	0.73	0.06	0.07	46	1852	Senkyunolide F	0.18	0.10	0.01	0.01
19	1443	β-Linalool	0.79	0.47	0.07	0.07	47	1866	Hexadecanoic acid	0.01	0.02	0.06	0.03
20	1448	3-Butylidene-4-hydroxyphthalide	0.23	0.12	0.05	0.04	48	1869	1-Octadecanol	0.20	0.13	0.13	0.07
21	1452	α,p-Dimethylstyrene	0.38	0.30	0.04	0.03	49	1898	Methoxsalen	0.22	0.21	0.07	0.05
22	1458	Cis-1,2-limonene epoxide	-	-	0.36	0.31	50	2022	Ledene	-	-	0.30	0.26
23	1461	2,5-Di-tert-Butylaniline	0.93	1.37	0.13	0.13	51	2052	Marmesin	0.21	0.21	0.05	0.04
24	1466	Lignocerol	0.12	0.24	0.03	0.02	52	2085	Lomatin	0.70	0.82	0.10	0.08
25	1470	4,8-Epoxyterpinolene	0.55	0.30	0.05	0.04	53	2095	Pentadecanoic acid	-	-	0.03	0.02
26	1474	7-Hexyltridecan-1-ol	0.49	0.27	0.07	0.06	54	2201	Linoleic acid	0.37	0.19	-	-
27	1479	β-Funebrene	0.62	0.32	0.05	0.05	55	2225	Methyl palmitate	0.13	0.07	-	-
28	1489	1-Phenyl-1-pentanone	-	-	0.02	0.01	56	2282	1-Pentadecanol	-	-	0.05	0.05

^*1*^*RI* Retention indices calculated against *n*-alkanes.

^*2*^*RA* Relative percentages to the total peak areas in the original crude herb. A total of 56 chemicals were identified, and the unknown peaks covered 4% in ASR and 1% in CR (*n*=5).

^*3*^*ASR* Angelicae Sinensis Radix, *WASR* Wine-treated Angelicae Sinensis Radix, *CR* Chuanxiong Rhizoma, *WCR* Wine-treated Chuanxiong Rhizoma.

^*4*^Target chemicals.

ASR and CR both belong to the Apiaceae family, and are quite similar in their chemical constituents, including phthalides (*e.g.*, Z-ligustilide, senkyunolide A, butylphthalide and Z-butylidenephthalide) and phenolic compounds (*e.g.*, ferulic acid and coniferyl ferulate) [18]. Wine treatment of ASR and CR is a commonly adopted procedure. In our findings, the changes of volatile chemicals in ASR and CR were distinctive after wine treatment. The amounts of β-pinene, β-myrcene and caryophyllene were reduced in wine-treated ASR, while these amounts were increased in wine-treated CR. Meanwhile, the amounts of Z-butylidenephthalide and Z-ligustilide were reduced in wine-treated ASR, but remained unchanged in wine-treated CR. Since wine treatment can reduce the amounts of volatile chemicals in ASR and CR, the chemical changes of these herbs would exhibit very different chemical profiles. We speculate that these differences may arise through differences in the cellular structures, tissue organizations and chemical compositions of the herbs.

### PCA of the GC-MS/MS fingerprints and chemical markers

**Figure 2:**
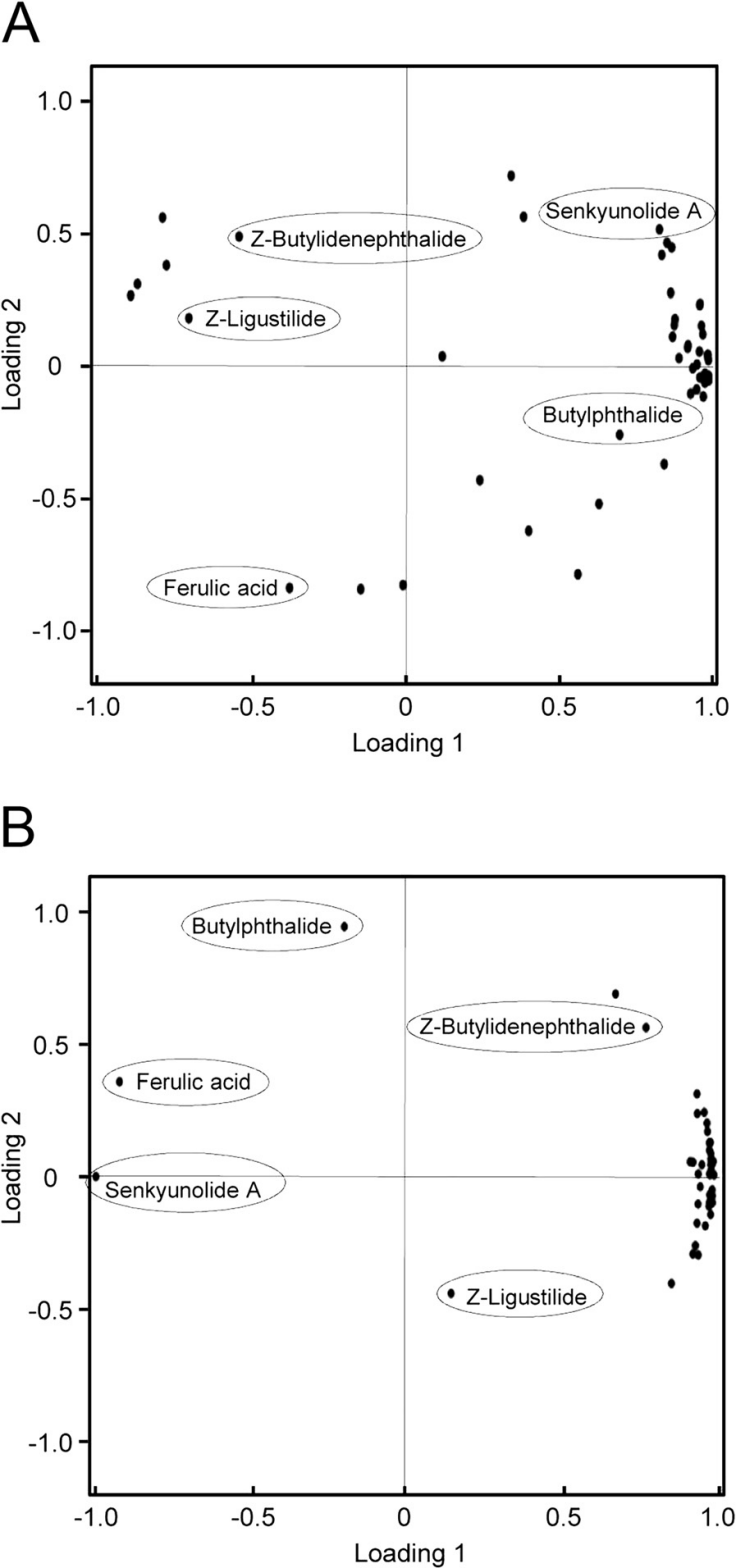
**Loading plots of PCA for ASR and CR.** (**A, B**) Loading plots for crude and wine-treated ASR (**A**) and crude and wine-treated CR (**B**) using common components as the input data. The PCA was performed on the relative peak areas using SPSS for Windows 16.0 software.

PCA provides a roadmap that shows how a complex data set can be transformed to a lower dimension to reveal some hidden, but important, chemical components [19]. In PCA, each data point represents a chemical (variable). The loading plots of PCA can reveal whether the chemical components contribute significantly to the intergroup differences in which they are farthest from the main cluster of analyzed chemicals. Ferulic acid, butylphthalide, Z-butylidenephthalide, senkyunolide A and Z-ligustilide were found to be distinctive from the main cluster profiles of ASR and CR, and as a result, these chemicals could be significant for discriminating crude ASR from wine-treated ASR (Figure 2A). Furthermore, the loading plots revealed that ferulic acid, butylphthalide and senkyunolide A were distinctive ingredients for the discrimination of wine-treated CR from crude CR (Figure 2B).

**Figure 3:**
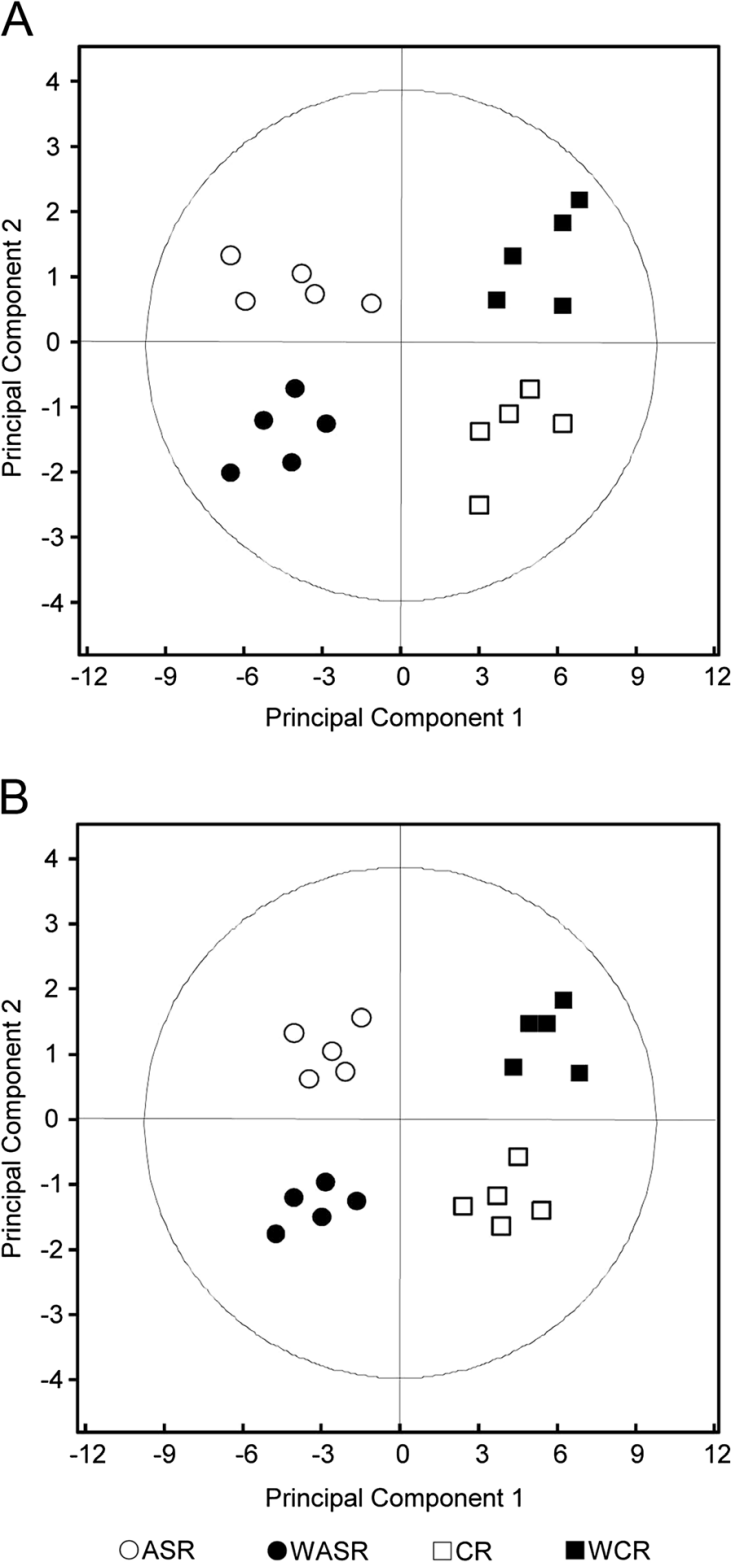
**PCA projection plots for ASR and CR.** (**A**) Score plots for crude ASR, wine-treated ASR, crude CR and wine-treated CR using common components as the input data. (**B**) Score plots for crude and wine-treated ASR and CR, as in (**A**), using the peak areas of five chemical markers, namely ferulic acid, butylphthalide, Z-butylidenephthalide, senkyunolide A and Z-ligustilide.

Application of PCA to the chromatographic fingerprints summarized the vast amount of GC-MS/MS data into principal component 1 (PC1) and principal component 2 (PC2), with two-dimensional score plots showing clear separation among different samples. PC1 and PC2 provided significant indications of >90% in total variability. The extracts of ASR and CR had similar compositions of volatile oils. Using GC fingerprinting of the volatile chemicals, PCA was applied to classify ASR and CR. ASR was characterized by negative scores for PC1, but positive scores for PC2, while CR had positive scores for PC1, but negative scores for PC2 (Figure 3A). After wine treatment, the volatile chemicals were modified in both ASR and CR, and resulted in a clearer classification between wine-treated ASR and wine-treated CR. Wine-treated ASR was characterized by negative scores for both PC1 and PC2, while wine-treated CR showed positive scores for PC1 and PC2. Therefore, GC-QQQ-MS/MS with application of pattern recognition could successfully differentiate crude and wine-treated ASR and CR by good indications of their chemical modifications.

## Conclusion

Different chemical profiles of ASR and CR after wine treatment could be identified by GC-QQQ-MS/MS. Five marker chemicals analyzed by PCA, namely ferulic acid, butylphthalide, Z-butylidenephthalide, senkyunolide A and Z-ligustilide, were sufficient to distinguish the crude and corresponding wine-treated forms of ASR and CR.

## Abbreviations

ASR: Angelicae Sinensis Radix; CR: Chuanxiong Rhizoma; PCA: Principal component analysis; GC-MS/MS: Gas chromatography coupled with tandem mass spectrometry; GC-QQQ-MS/MS: Gas chromatography-triple quadrupole mass spectrometry.

## Competing interests

The authors declare that they have no competing interests.

## Authors’ contributions

KT, DL, HL, RC and TD designed the study. JZ performed the experiments and wrote the manuscript. KZ, WZ, KZ, JC and PC helped to analyze the data. All authors read and approved the final version of the manuscript.

## Supplementary Material

Additional file 1 **MS fingerprint for five chemical markers in Figure** 1**.** GC-MS fingerprint chromatograms of five chemical markers: ferulic acid, butylphthalide, Z-butylidenephthalide, senkyunolide A and Z-ligustilide. One μL was injected. The information for each chemical was indicated. Click here for file

Additional file 2: Table S1 Calibration curves, LOD and LOQ for marker chemicals. Click here for file

Additional file 3: Table S2 Precision, repeatability and recovery of marker chemicals in Angelicae Sinensis Radix. Click here for file
