# Supplementary figures and images for: Chemical changes of Angelicae Sinensis Radix and Chuanxiong Rhizoma by wine treatment: chemical profiling and marker selection by gas chromatography coupled with triple quadrupole mass spectrometry
Source: Chin Med. 2013 Jun 6;8:12. doi: 10.1186/1749-8546-8-12 (PMC3693868; doi:10.1186/1749-8546-8-12)

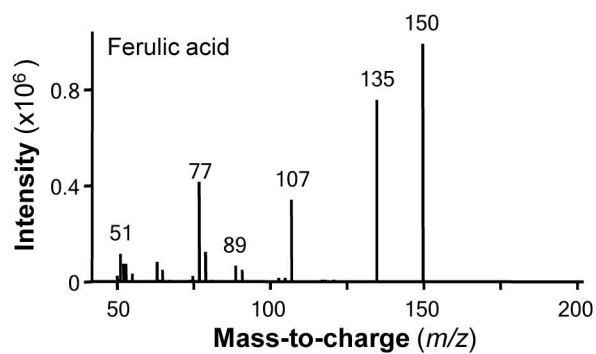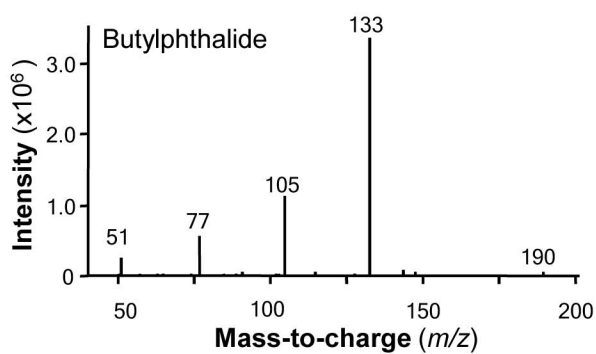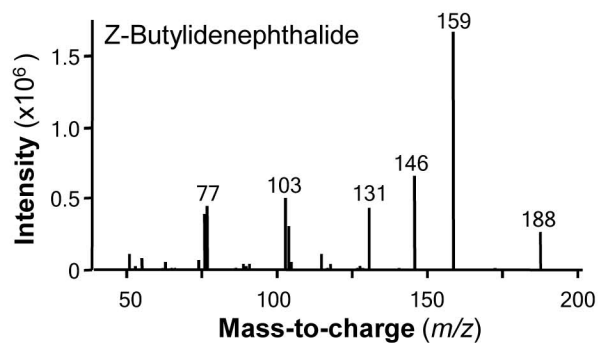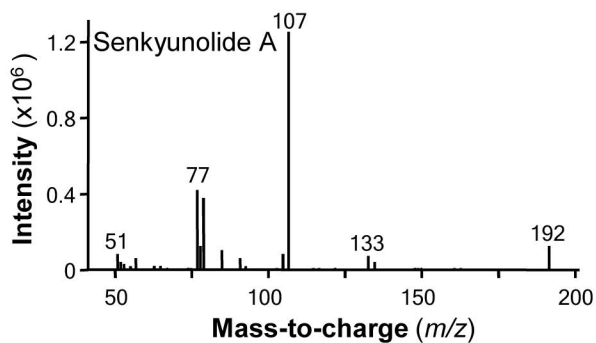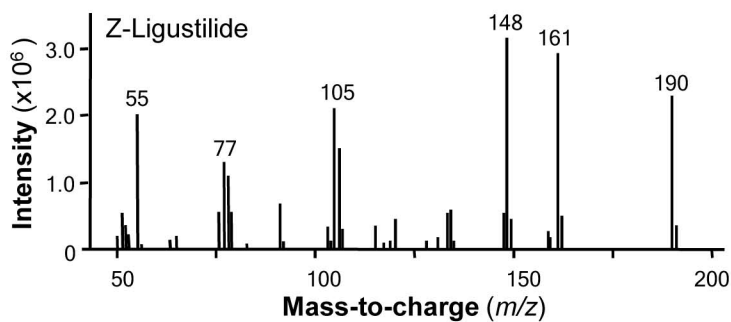

Supplement: Additional file 1 — MS fingerprint for five chemical markers in Figure 1. GC-MS fingerprint chromatograms of five chemical markers: ferulic acid, butylphthalide, Z-butylidenephthalide, senkyunolide A and Z-ligustilide. One μL was injected. The information for each chemical was indicated. [file 1749-8546-8-12-S1.pdf]
