# Supplementary material for: Chemical changes of Angelicae Sinensis Radix and Chuanxiong Rhizoma by wine treatment: chemical profiling and marker selection by gas chromatography coupled with triple quadrupole mass spectrometry
Source: Chin Med. 2013 Jun 6;8:12. doi: 10.1186/1749-8546-8-12 (PMC3693868; doi:10.1186/1749-8546-8-12)
Supplement: Additional file 2: Table S1 — Calibration curves, LOD and LOQ for marker chemicals. [file 1749-8546-8-12-S2.doc]

**Supplementary Table 1. Calibration curves, LOD and LOQ for marker chemicals**

| **No.** | **Chemical** | **Calibration curve** 1 | **Correlation factor** (*r*2) | **Linearity**  (µg/mL) | **LOD** 2  (µg/mL) | **LOQ** 3  (µg/mL) |
| --- | --- | --- | --- | --- | --- | --- |
| 1 | **Ferulic acid** | Y = 143.2X – 74587.4 | 0.9985 | 0.1~5 | 0.02 | 0.08 |
| 2 | **Butylphthalide** | Y = 3599.5X – 38126.2 | 0.9987 | 0.02~0.1 | 0.001 | 0.005 |
| 3 | **Z-Butylidenephthalide** | Y = 1556.1X – 96984.3 | 0.9986 | 0.02~2 | 0.002 | 0.006 |
| 4 | **Senkyunolide A** | Y = 1047.4X – 26114.7 | 0.9962 | 0.02~1 | 0.003 | 0.011 |
| 5 | **Z-Ligustilide** | Y = 85.8X – 3215.4 | 0.9932 | 0.1~1 | 0.02 | 0.07 |

*^1^*The calibration curves were constructed by plotting the peak area versus the concentration of each analyte. Each calibration curve was derived from six data points (*n*=3).

*^2^*LOD refers to the limit of detection (S/N=3).

*^3^*LOQ refers to the limit of quantification (S/N=10).
