# Supplementary material for: Chemical changes of Angelicae Sinensis Radix and Chuanxiong Rhizoma by wine treatment: chemical profiling and marker selection by gas chromatography coupled with triple quadrupole mass spectrometry
Source: Chin Med. 2013 Jun 6;8:12. doi: 10.1186/1749-8546-8-12 (PMC3693868; doi:10.1186/1749-8546-8-12)
Supplement: Additional file 3: Table S2 — Precision, repeatability and recovery of marker chemicals in Angelicae Sinensis Radix. [file 1749-8546-8-12-S3.doc]

**Supplementary Table 2. Precision, repeatability and recovery of marker chemicals in Angelicae Sinensis Radix**

| **Chemical** | **Precision** | | | | **Repeatability** (*n*=5) | | **Recovery** 1 (*n*=3) | |
| --- | --- | --- | --- | --- | --- | --- | --- | --- |
|  | **Intra-day** 2 (*n*=5) | | **Inter-day** 3 (*n*=6) | |  | |  | |
|  | **Mean** (µg/mL) | **RSD**  (%) | **Mean** (µg/mL) | **RSD**  (%) | **Mean** (µg/mL) | **RSD**  (%) | **Mean** (%) | **RSD**  (%) |
| **Ferulic acid** | 1.341 | 2.52 | 1.363 | 3.16 | 1.328 | 1.34 | 96.29 | 4.13 |
| **Butylphthalide** | 0.021 | 3.27 | 0.015 | 3.72 | 0.021 | 2.01 | 95.58 | 3.56 |
| **Z-Butylidenephthalide** | 0.078 | 3.54 | 0.071 | 4.65 | 0.066 | 2.42 | 98.22 | 3.14 |
| **Senkyunolide A** | 0.149 | 4.13 | 0.156 | 2.85 | 0.151 | 3.03 | 97.01 | 2.73 |
| **Z-Ligustilide** | 1.277 | 2.35 | 1.221 | 2.12 | 1.198 | 1.57 | 98.75 | 1.55 |

*^1^*Recovery (%) = 100 × (selected amount – original amount) / amount spiked. The data were presented as the averages of three independent determinations, and the SD was <5% of the mean. In the table, ASR was used as a matrix for analysis. The results using CR as a matrix were similar.

*^2^*The intra-day analysis refers to a sample examined as five replicates within 1 day.

*^3^*The inter-day analysis refers to a sample examined in duplicate over 3 consecutive days.
